# Obsessive Belief Dimensions as Predictors of OCD Symptom Dimensions in Different Trauma Types

**DOI:** 10.1192/j.eurpsy.2026.10641

**Published:** 2026-07-31

**Authors:** E. Karakaya, Z. Çakır

**Affiliations:** 1 Psychology, Karadeniz Technical University, Trabzon; 2 Psychology, Hacettepe University, Ankara, Türkiye

## Abstract

**Introduction:**

Obsessive beliefs are considered one of the main etiological factors of Obsessive-Compulsive Disorder (OCD) within cognitive models. More specifically, it has been suggested that different obsessive belief dimensions may be more strongly associated with some OCD symptom dimensions. However, mixed findings on these relationships have been reported, and environmental factors have not been adequately considered.

**Objectives:**

In the present study, it was examined which obsessive belief dimensions predict which OCD symptom dimensions across different traumatic experience groups. Accordingly, the aim was to elaborate on these relationships within the framework of specific environmental factors, thereby contributing to the understanding of cognitive models of OCD and clarifying the mixed findings in the literature.

**Methods:**

The sample consisted of 395 participants aged 18–59 years (*M* = 25.10, *SD* = 6.96) who reported at least one traumatic experience. The Obsessive-Compulsive Inventory Revised Form (OCI-R) was used to measure OCD symptoms, and the Obsessive Beliefs Questionnaire-20 (OBQ-20) was used to measure obsessive beliefs. Participants were categorized into five distinct groups based on the most disturbing traumatic experience they reported. In each trauma group, a series of two-step hierarchical regression analyses was performed, with each OCD symptom dimension as the outcome variable. In these analyses, age, the total score of depression and anxiety, and the total score of adverse childhood experiences were included in the first step; obsessive belief dimensions were entered in the second step.

**Results:**

In the life-threatening illness trauma group, importance and control of thoughts significantly predicted obsessing (*β* = .28, *p* = .029). In the accident trauma group, overestimation of threat significantly predicted ordering (*β* = .90, *p* = .003), and importance and control of thoughts significantly predicted neutralizing (*β* = .59, *p* = .019). In the natural disaster trauma group, perfectionism significantly predicted checking (*β* = .35, *p* = .040); importance and control of thoughts significantly predicted washing (*β* = .29, *p* = .023); and both perfectionism (*β* = .37, *p* = .022) and importance and control of thoughts (*β* = .31, *p* = .013) significantly predicted neutralizing. In the bereavement and interpersonal trauma groups, none of the obsessive belief dimensions significantly predicted any specific OCD dimension.

**Conclusions:**

Through the two-step hierarchical regression analyses, it was revealed that the relationships between obsessive beliefs and OCD symptoms showed different patterns across the trauma groups. Within this scope, the current findings suggest that it may be important to consider the type of traumatic experience in the etiology and treatment of OCD. Future studies should examine in greater detail which characteristics of traumatic experiences may account for differences in these relationships.

**Disclosure of Interest:**

None Declared

